# A randomized controlled trial of an app-based intervention on physical activity and glycemic control in people with type 2 diabetes

**DOI:** 10.1186/s12916-024-03408-w

**Published:** 2024-05-01

**Authors:** Gyuri Kim, Seohyun Kim, You-Bin Lee, Sang-Man Jin, Kyu Yeon Hur, Jae Hyeon Kim

**Affiliations:** 1 Division of Endocrinology and Metabolism, Department of Medicine, Samsung Medical Center, Sungkyunkwan University School of Medicine, 81, Irwon-Ro, Gangnam-Gu, Seoul, 06351 Republic of Korea; 2 Department of Clinical Research Design and Evaluation, Samsung Advanced Institute for Health Sciences and Technology, Sungkyunkwan University, Seoul, 06355 Republic of Korea

**Keywords:** Physical activity, Type 2 diabetes, Application, Text messages, Smartphone, Step

## Abstract

**Background:**

We investigated the effects of a physical activity encouragement intervention based on a smartphone personal health record (PHR) application (app) on step count increases, glycemic control, and body weight in patients with type 2 diabetes (T2D).

**Methods:**

In this 12-week, single-center, randomized controlled, 12-week extension study, patients with T2D who were overweight or obese were randomized using ratio 1:2 to a group using a smartphone PHR app (control group) or group using the app and received individualized motivational text messages (intervention group) for 12 weeks. During the extension period, the sending of the encouraging text messages to the intervention group was discontinued. The primary outcome was a change in daily step count after 12 weeks and analyzed by independent t-test. The secondary outcomes included HbA1c, fasting glucose, and body weight analyzed by paired or independent t-test.

**Results:**

Of 200 participants, 62 (93.9%) and 118 (88.1%) in the control and intervention group, respectively, completed the 12-week main study. The change in daily step count from baseline to week 12 was not significantly different between the two groups (*P* = 0.365). Among participants with baseline step counts < 7,500 steps per day, the change in the mean daily step count at week 12 in the intervention group (1,319 ± 3,020) was significantly larger than that in control group (-139 ± 2,309) (*P* = 0.009). At week 12, HbA1c in the intervention group (6.7 ± 0.5%) was significantly lower than that in control group (6.9 ± 0.6%, *P* = 0.041) and at week 24, changes in HbA1c from baseline were significant in both groups but, comparable between groups. Decrease in HbA1c from baseline to week 12 of intervention group was greater in participants with baseline HbA1c ≥ 7.5% (-0.81 ± 0.84%) compared with those with baseline HbA1c < 7.5% (-0.22 ± 0.39%) (P for interaction = 0.014). A significant reduction in body weight from baseline to week 24 was observed in both groups without significant between-group differences (*P* = 0.370).

**Conclusions:**

App-based individualized motivational intervention for physical activity did not increase daily step count from baseline to week 12, and the changes in HbA1c levels from baseline to week 12 were comparable.

**Trial registration:**

ClinicalTrials.gov (NCT03407222).

**Supplementary Information:**

The online version contains supplementary material available at 10.1186/s12916-024-03408-w.

## Background

It is anticipated that the global prevalence of diabetes will rise from 10.5% (536.6 million people) in 2021 to 12.2% (783.2 million people) in 2045. Additionally, the cost of treating and controlling diabetes and its related microvascular and macrovascular complications is estimated to increase from $966 billion in 2021 to $1,054 billion in 2045 [1]. Exercise has long been recognized as important in the management and treatment of metabolic diseases such as diabetes, metabolic syndrome, and obesity [2, 3]. The American Diabetes Association recommends that people with diabetes engage in at least 150 min of moderate-intensity aerobic activity, such as walking three–seven days a week, with no more than two days off [4]. Previous studies have also reported that walking more than 6500 steps per day is helpful in treating chronic diseases [5]. Also, 8,000–10,000 steps per day are recommended, approximately equivalent to walking for 60 min at an intensity of three metabolic equivalents of tasks (METs) per day, which corresponds to 23 METs-hours of moderate physical activity per week [5]. Regular exercise has a beneficial effect on blood glucose control, weight loss, and insulin resistance and has been reported to be associated with a decrease in overall mortality and cardiovascular mortality in type 2 diabetes [6–9]. However, few randomized controlled trials have investigated whether step count interventions affect step count, blood glucose levels, and body weight in patients with well-controlled type 2 diabetes. A previous study of systematic review and meta-analyses found that using apps can improve lifestyle aspects and reduce HbA1c levels in patients with diabetes [10]. A recent randomized controlled trial investigated an app-based lifestyle intervention to increase physical activity in patients with type 2 diabetes, but the app was only provided to the intervention group [11]. App-based intervention did not increase physical activity over 52 weeks compared with control group, although apparent benefits were observed for physical-related quality of life. The difference between the two groups may be attributed not only to the intervention effect of the app but also to the use of the app itself. Recent research on digital treatment showed that the intervention group received a device with active software designed to treat a specific disease, while the control group also received a device with inactive software, to control for the potential effects of the software or the device itself, similar to the placebo effect observed in drug trials [12]. Therefore, in this study, we monitored step count using a smartphone personal health record (PHR) application (app) and sent text messages encouraging physical activity once a week for 12 weeks to evaluate the effect of this intervention on step count increase, glycemic control, and body weight for 12 weeks and an extension period of 12 weeks in patients with well-controlled type 2 diabetes.

## Methods

This was a single-center, double-arm, open-label, 12-week randomized controlled trial with a 12-week extension study comparing two groups: 1) that used a smartphone PHR app (control group) and 2) that used the app and received individualized motivational text messages every week to increase daily step counts based on the mean number of steps collected per day by the app (intervention group). The trial was conducted at the Diabetes Center, Division of Endocrinology and Metabolism of the Samsung Medical Center (SMC), Republic of Korea, and participants were recruited from April 2018 to September 2019. All the participants provided written informed consent. The Institutional Review Board (IRB) of the SMC approved the study protocol (No. 2017–12-061), which was in accordance with the ethical guidelines of the Declaration of Helsinki and Korea Good Clinical Practice. This study was registered at ClinicalTrials.gov (NCT03407222).

### Study participants

Eligibility criteria included patients with type 2 diabetes aged 20–69 years, with a HbA1c of less than 8.5%, who have not taken anti-diabetes medication for the past 4 weeks or who have taken more than or equal to one oral hypoglycemic agent for more than 12 weeks using the same dosage, who had overweight or obesity (body mass index (BMI) ≥ 23 kg/m^2^), who were able to use an Android smartphone and wireless internet, and who voluntarily agreed to participate. The exclusion criteria were as follows: diabetes other than type 2 diabetes, including type 1 diabetes and gestational diabetes; use of insulin or a GLP-1 receptor agonist; presence of comorbidities such as uncontrolled chronic liver disease, acute kidney injury, and psychological disorders; use of a weight-lowering agent; presence of alcohol or drug addiction within the previous 3 years; use of systemic corticosteroids; pregnancy or lactation; no voluntary agreement to participate in the study; and unsuitable for participating in clinical research.

### Study design

This trial consisted of the following three periods: a one-week run-in period, a 12-week randomized treatment period, and a 12-week extension period (Figure S1). At visit 1 (week -1), the participants who met the inclusion criteria without meeting any exclusion criteria were provided smartphone PHR app to record blood glucose, blood pressure, and body weight, developed by Samsung Medical Center. The step count was measured using the Samsung Health application, which was automatically linked to the PHR app. The number of steps taken by all participants was automatically uploaded to the app, and the weight measured using the Bluetooth scale provided to all participants was continuously linked to the app. The baseline number of steps was measured during the one-week run-in-period. At week 0, the participants were assigned randomly at 2:1 ratio to the intervention or control group for 12 weeks. During the 12-week period, both groups used the smartphone PHR app, and the intervention group received text messages every week, which encouraged step-by-step increments according to the mean daily number of steps per week monitored by the app, whereas the control group did not receive text messages. The participants of intervention groups were divided into six groups such as 1) basal activity (< 2,500 steps/day), 2) limited activity (2,500–4,999 steps/day), 3) low activity (5,000–7,499 steps/day), 4) somewhat active (7,500–9,999 steps/day), 5) active (10,000–12,499 steps/day), 6) highly active (≥ 12,500 steps/day) according to the average number of steps per day. [13]). The average number of steps taken by each participant per day during the week was calculated and divided them into five groups. We suggested a step goal based on participants’ average number of steps in the past week. If a participant took an average of 9,000 steps per day during the week, a message was sent: 'You walked less than 10,000 steps a day on average this week. Next week, aim to walk more than 10,000 steps a day! Making changes to your lifestyle can improve your health.' During the extension period, for 12 weeks from week 12 to week 24), encouraging text messages were discontinued in the intervention group to check the durability of the intervention.

### Data collection

Demographic, anthropometric, and laboratory data were collected from all participants. Body weight and height were measured, and the BMI was estimated as body weight (kg) divided by height squared (*m*^2^). Smoking status and alcohol consumption data were collected using a self-reported questionnaire and classified as never, ever, or current, and either less than a cup per day or not, respectively. The estimated glomerular filtration rate (eGFR) was estimated using the Chronic Kidney Disease Epidemiology Collaboration 2021 (CKD-EPI 2021) formula [14]. Medical history data, including duration of diabetes and type of medication, were collected. During the study period, the average step count per week, laboratory data, body weight, and physical activity were obtained at weeks 12 and 24 in both groups. Physical activity was measured using the 7-item International Physical Activity Questionnaire-Short Form (IPAQ-SF) designed by a team of specialists in physical activity for population-level tracking of adult physical activity [15, 16]. Physical activity was classified with the following three categories: walking, moderate physical activity, and vigorous physical activity [17]. We used the total Metabolic Equivalent of Task (MET)–min/week to express weekly metabolic engagement in walking and in both moderate and vigorous physical activity practice.

### Outcomes

The primary outcome was the change in daily step count between the control and intervention groups after 12 weeks of intervention. Secondary outcomes included e mean daily step count at weeks 12 and 24, mean HbA1c levels at weeks 12 and 24, fasting glucose levels at weeks 12 and 24, body weight at weeks 12 and 24, physical activity at weeks 12 and 24, and lipid levels including total cholesterol, low-density lipoprotein (LDL) cholesterol, high-density lipoprotein (HDL) cholesterol, and triglycerides at weeks 12 and 24.

### Statistical analysis

Assuming a 1,000 step difference in daily step count increments between the study groups (standard deviation, 2,150 daily steps) according to previous studies [18–20], a sample size of 55 and 111 participants for control and intervention group, respectively was needed for a two-sided alpha threshold of 0.05 and 80% power. We aimed to recruit 200 participants to allow for a dropout rate of 17% during follow-up (control group, *n* = 66; intervention group, *n* = 134).

Continuous and categorical variables are summarized as mean and standard deviation (SD) and percentages, respectively. Statistical analysis of the primary outcome was performed according to the intention-to-treat principle in the full analysis set (FAS) (i.e., all randomized participants used the smartphone PHR app and had baseline measurements and at least one measurement during the study period). Two-sample t-tests for continuous variables and chi-square tests for categorical variables were used to compare the baseline characteristics between the intervention and control groups. For the primary outcome, we conducted a two-sample t-test for the difference in the mean change in the daily step count between the intervention and control groups after 12 weeks. For secondary outcomes, we conducted independent t-tests for the mean difference at 12 and 24 weeks and change in mean difference from baseline to 12 and 24 weeks in average steps per day, HbA1c, fasting glucose, body weight, and total MET-min/week. A paired t-test was used to assess the mean difference in the intervention and control groups from baseline to 12 and 24 weeks. Although it is not pre-specified, subgroup analyses were conducted in the FAS, stratified by the baseline HbA1c levels (< 7.5% and ≥ 7.5%) and baseline daily step counts (< 7,500 and ≥ 7,500 steps). The mean HbA1c level for the entire population was 7.1% and to differentiate between low and high HbA1c groups with appropriate sample sizes, a cut-off value of 7.5% was chosen (*n* = 152 vs *n* = 30). A previous study conducted on the Asian population found that taking more than 7,500 steps per day was associated with a decrease in BMI and body fat, compared to taking fewer than 7,500 steps [13]. To identify the differences in outcomes by group and time, we conducted a linear mixed effect model for step counts and hemoglobin A1c considering random slope and intercept. All statistical analyses were performed using R software, version 4.1.3 (R Foundation for Statistical Computing),. Two-sided *P* values < 0.05 were regarded as significant.

## Results

### Baseline characteristics

**Fig. 1:**
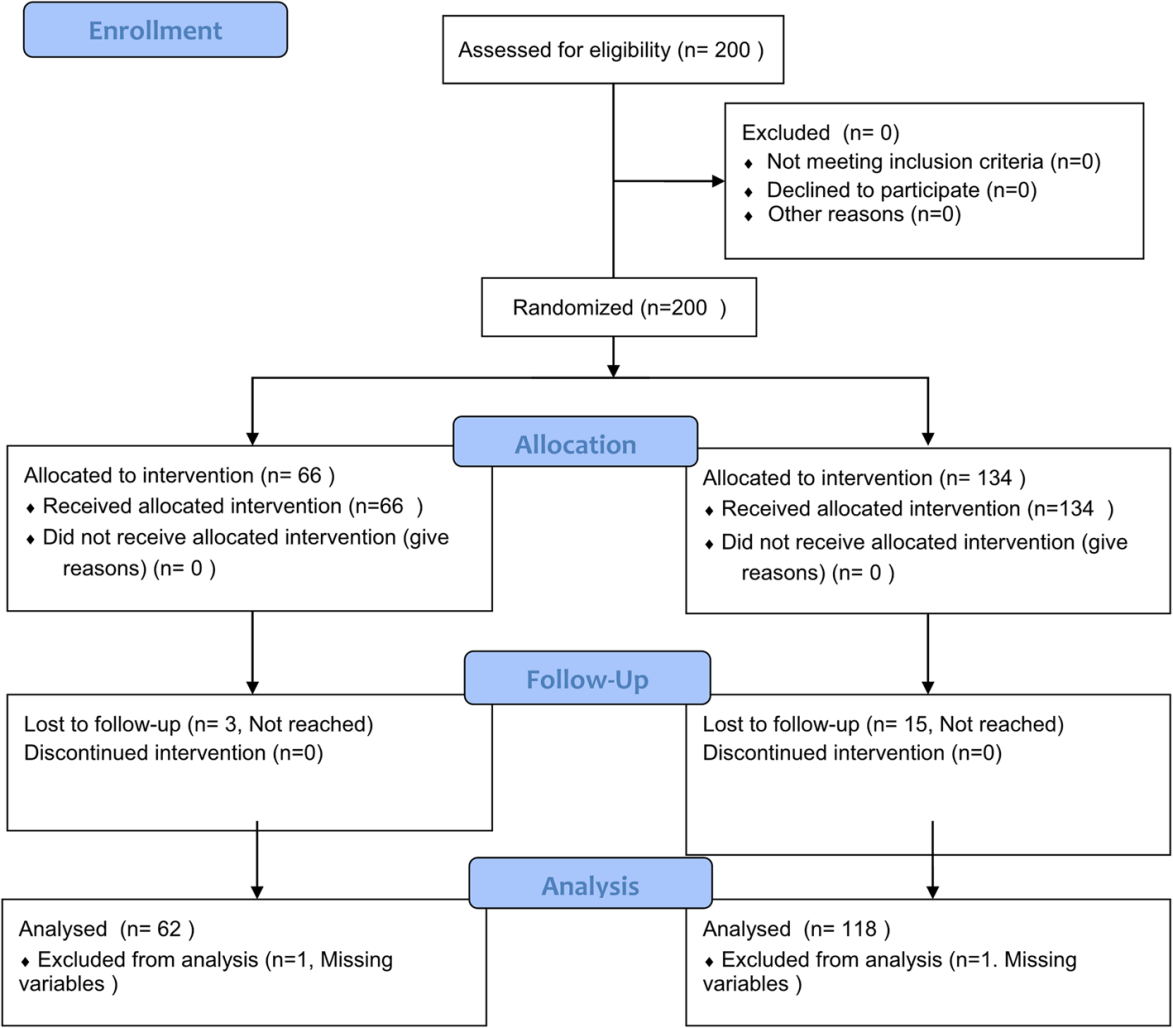
CONSORT flow diagram

Of the 200 participants, 66 and 134 were randomly assigned to the control and intervention groups, respectively. Of the randomized participants, 63 (95.5%) in the control group and 119 (88.8%) in the intervention group completed the 12-week main study (Fig. 1, Figure S1). Among them, 63 participants in the control group and 119 participants in the intervention group participated in the extension study as the control/control and intervention/control groups, respectively. Finally, 63 participants (95.5%) in the control/control group and 119 participants (88.8%) in the intervention/control group completed the 12-week extension study.

The baseline characteristics of the study participants on the FAS are summarized in Table 1. The baseline characteristics of the intervention and control groups were similar, with comparable age, sex, BMI, duration of diabetes, level of physical activity, use of oral antidiabetic agents, and HbA1c levels. The mean age of the study participants was 57.5 ± 6.8 years, 54 (29.7%) of participants were female, and mean duration of diabetes was 6.8 ± 6.1 years. The mean baseline BMI was 26.5 ± 2.6 kg/m^2^ and the mean baseline HbA1c level was 7.1 ± 0.4%.

**Table 1:** Baseline characteristics of the study participants of the full analysis set

	**Control (*N* = 63)**	**Intervention (*N* = 119)**	***P* value**
Age, years	58.3 ± 5.8	57.1 ± 7.2	0.257
Sex			0.684
Female	17 (27.0%)	37 (31.1%)	
Male	46 (73.0%)	81 (68.9%)	
Weight, kg	73.5 ± 8.6	73.7 ± 9.8	0.898
Body Mass Index, kg/m^2^	26.5 ± 2.8	26.5 ± 2.6	0.966
Systolic Blood Pressure, mmHg	128.8 ± 15.8	127.5 ± 14.3	0.586
Diastolic Blood Pressure, mmHg	75.2 ± 10.2	75.1 ± 10.2	0.967
Smoking status			0.721
Never	48 (76.2%)	92 (77.3%)	
Ever	8 (12.7%)	11 (9.2%)	
Current	7 (11.1%)	16 (13.4%)	
Alcohol consumption			0.539
< 1 cup per a day	36 (57.1%)	75 (63.0%)	
≥ 1 cup per a day	27 (42.9%)	44 (37.0%)	
Family history of diabetes			0.952
No	36 (57.1%)	70 (58.8%)	
Yes	27 (42.9%)	49 (41.2%)	
Medication for hypertension			0.57
No	33 (52.4%)	69 (58.0%)	
Yes	30 (47.6%)	50 (42.0%)	
Lipid modifying agents			0.519
No	9 (14.3%)	23 (19.3%)	
Yes	54 (85.7%)	96 (80.7%)	
Duration of diabetes, years	7.0 ± 6.0	6.7 ± 6.2	0.818
Level of Physical Activity^a^			0.568
High	17 (27.0%)	24 (20.3%)	
Moderate	13 (20.6%)	29 (24.6%)	
Low	33 (52.4%)	65 (55.1%)	
Oral antidiabetic agents, yes	43 (68.3%)	79 (66.4%)	0.929
Metformin	42 (66.7%)	77 (64.7%)	
Sulfonylurea	13 (20.6%)	17 (14.3%)	
DPP4 inhibitor	11 (17.5%)	30 (25.2%)	
Thiazolidinedione	3 (4.8%)	8 (6.7%)	
SGLT2 inhibitor	13 (20.6%)	21 (17.6%)	
HbA1c, %	7.1 ± 0.4	7.0 ± 0.4	0.53
Fasting glucose, mg/dL	135.2 ± 21.1	138.2 ± 24.9	0.421
Total cholesterol^b^, mg/dL	142.8 ± 27.2	147.6 ± 29.4	0.281
HDL cholesterol^c^, mg/dL	55.1 ± 14.1	50.6 ± 13.7	0.036
Triglyceride^c^, mg/dL	131.1 ± 63.7	154.8 ± 140.7	0.123
LDL cholesterol^c^, mg/dL	81.8 ± 25.6	86.7 ± 28.7	0.256
Creatinine^d^, mg/dL	0.8 ± 0.2	0.9 ± 0.2	0.448
eGFR^d^*,* ml/min/1.73 m^2^	96.4 ± 11.1	95.1 ± 12.5	0.49

*DPP4* Dipeptidyl peptidase 4, *eGFR* estimated glomerular filtration rate, *HDL* high-density lipoprotein, *LDL* low-density lipoprotein, *SD* standard deviation, *SGLT2* Sodium/glucose cotransporter 2

Values are mean ± SD or n (%)

^a^One study with missing data

^b^Three with missing data

^c^Two with missing data

^d^Four with missing data

### Change in mean daily step count

**Fig. 2:**
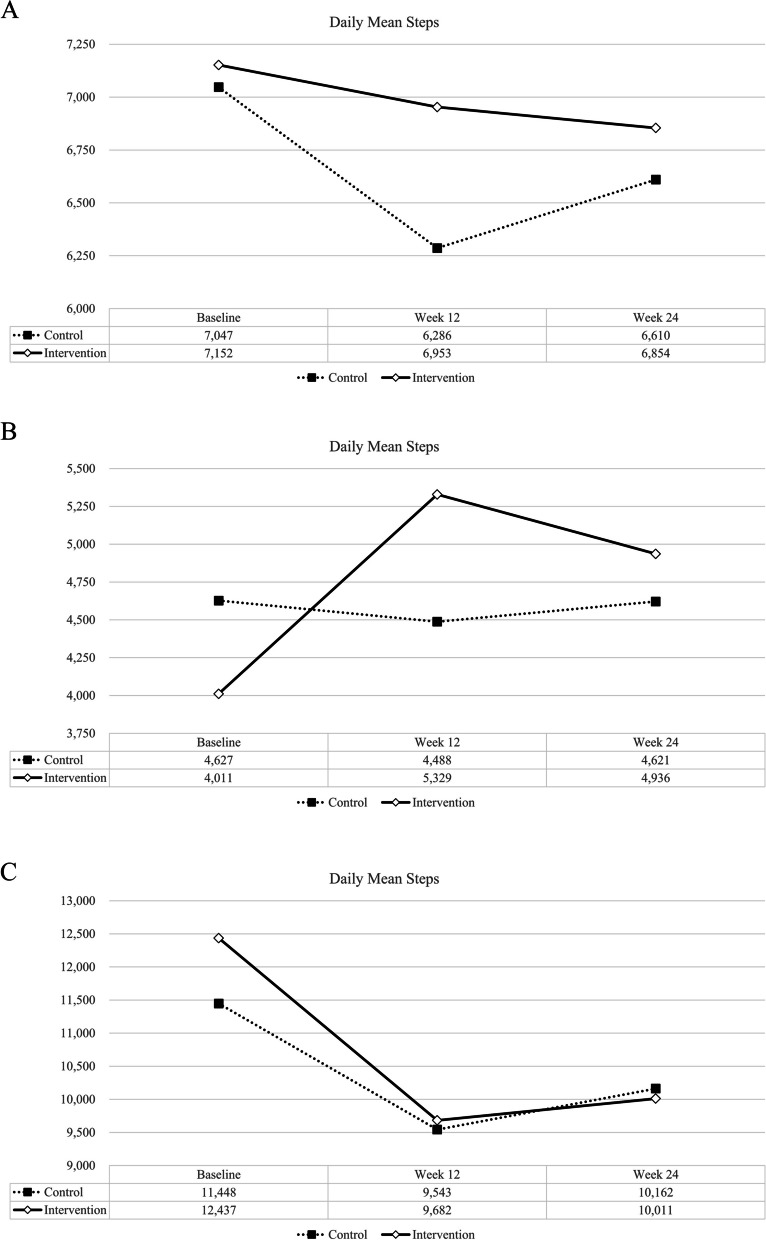
**A** Changes in mean step counts per a day. **2 B**. Changes in mean step counts per a day (participants with baseline daily average step < 7,500, *N* = 114). **C**. Changes in mean step counts per a day (participants with baseline daily average step ≥ 7,500, *N* = 66)

Changes in daily step count from baseline to weeks 12 and 24 are shown in Table 2 and Fig. 2A. At baseline, the mean step count per day was similar between the two group (*P* = 0.89). From baseline to week 12, the change in the daily step count was -766 ± 3,570 for the control group and -200 ± 4,160 for the intervention group, which was not significantly different between the two groups (*P* = 0.365). In addition, changes in mean step counts per day from baseline to week 24 were not significantly different between the two groups (*P* = 0.828); however, among participants with baseline mean daily step counts of less than 7,500 steps per day (*N* = 114), the increase in mean daily step count from baseline to week 12 in the intervention group (1,319 ± 3,020) was significantly larger than that in the control group (-139 ± 2,309), showing a significant between-group difference (*P* = 0.009) (Fig. 2B). In those with baseline mean daily step counts equal or more than 7,500 steps per a day (*N* = 66), there was no significant difference in the change of daily step count from baseline to week 12 between the two groups (*P* = 0.493) (Fig. 2C). In the linear mixed effect model, there was no significant interaction effect observed between group and time for step counts from baseline to week 24 (Table S1).

**Table 2:** Baseline, week 12, week 24, and changes in daily step counts from baseline

	**Control (*N* = 62)**	**Intervention (*N* = 118)**	***P* value**
Daily mean step at baseline	7047 ± 4348	7153 ± 5096	0.890
Daily mean step at week 12	6281 ± 3731	6952 ± 4054	0.280
Changes from baseline to week 12	-766 ± 3570	-200 ± 4160	0.365
Daily average step at week 24^a^	6653 ± 4370	6829 ± 4132	0.793
Changes from baseline to week 24^a^	-449 ± 3256	-324 ± 3817	0.828

Values are presented as the mean ± standard deviation

^a^Two with missing data in control group

### Glycemic outcomes

The mean HbA1c levels at weeks 12 and 24 and changes in HbA1c from baseline to weeks 12 and 24 in the control and intervention groups are shown in Table 3 and Figure S2. At week 12, HbA1c value in the intervention group (6.7 ± 0.5%) was significantly lower than that in the group (6.9 ± 0.6%, *P* = 0.043). The change in HbA1c from baseline to week 12 was -0.31 ± 0.53% for the intervention group (*P* < 0.001) and -0.18 ± 0.57% for the control group (*P* = 0.015) with no significant difference between the two groups (*P* = 0.167). In the subgroup analysis based on the baseline HbA1c level (< 7.5% and ≥ 7.5%) (Figures S3 and S4), the intervention group showed a greater decrease in HbA1c from baseline to week 12 in participants with a baseline HbA1c ≥ 7.5% (-0.81 ± 0.84% to week 12) compared to those with a baseline HbA1c < 7.5% (-0.22 ± 0.39% to week 12). This was not observed in the control group (-0.23 ± 1.04% with baseline HbA1c ≥ 7.5%, -0.17 ± 0.42% with baseline HbA1c < 7.5%) (P for interaction = 0.014). There was significant decrease in HbA1c from baseline to week 24 of -0.25 ± 0.58% in the intervention group (*P* < 0.001) compared to -0.15 ± 0.62% in the control group (*P* = 0.054) with no significant difference between the groups (*P* = 0.326). The decrease in fasting glucose from baseline to week 12 in the intervention group (-9.22 ± 21.25 mg/dL, *P* < 0.001) was significantly larger than in the control group (-0.84 ± 22.92 mg/dL, *P* = 0.774) with significant group difference (*P* = 0.016) (Table S2). There was no significant interaction effect between group and time for HbA1c from baseline to week 24 in the linear mixed effect model (Table S1).

**Table 3:** Changes in HbA1c from baseline to week 12 and week 24

	**Control**		**Intervention**		***P*-value**
	N	HbA1c (%)	N	HbA1c (%)	
Baseline (Mean ± SD)	63	7.1 ± 0.4	119	7.0 ± 0.4	0.53^b^
At week 12 (Mean ± SD)	63	6.9 ± 0.6	119	6.7 ± 0.5	0.043^b^
Changes from baseline to week 12 (Mean ± SD)	-0.18 ± 0.57		-0.31 ± 0.53		0.167^b^
*P*-value for mean difference from baseline to week 12	0.015^a^		< 0.001^a^		
At week 24 (Mean ± SD)	63	6.9 ± 0.6	118	6.8 ± 0.6	0.12^b^
Changes from baseline to week 24 (Mean ± SD)	-0.15 ± 0.62		-0.25 ± 0.58		0.326^b^
*P*-value for mean difference from baseline to week 24	0.054^a^		< 0.001^a^		

*Abbreviations*: *HbA1c* glycated hemoglobin, *SD* standard deviation

^a^*P*-values were derived using paired t-tests

^b^*P*-values were derived from a two-sample t-test

### Changes in body weight, physical activity, and lipid levels

The changes in body weight from baseline to weeks 12 and 24 in the control and intervention groups are shown in Table S3. In participants in the control group, changes in body weight were non-significant at week 12 (− 0.65 kg, *P* = 0.161) but significant at week 24 (− 1.05 kg, *P* = 0.03) compared with baseline. In participants in the intervention group, changes in body weight were significant at week 12 and 24 compared with baseline (− 1.07 kg and -1.46 kg, all *P* < 0.001). There were no significant differences between the two groups in terms of body weight from baseline to weeks 12 and 24. The total MET-min/week was similar between the two groups at baseline, week 12, and week 24 (Table S4)**.** Lipid levels including total cholesterol, LDL cholesterol, HDL cholesterol, and triglycerides, were similar between the two groups at week 12, and week 24 (Table S5).

## Discussion

In this 12-week, single-center, randomized, open-label, controlled, and 12-week extension trial involving individuals with type 2 diabetes who had HbA1c < 8.5% and BMI ≥ 23 kg/m^2^, all participants used smartphone PHR app. The intervention group additionally received individualized motivational text messages to increase daily step counts based on the information collected by app for the first 12-week periods. Although changes in the daily step count from baseline to week 12 between the intervention and control groups were not significantly different, there was a between-group difference in participants with a baseline mean daily steps of less than 7,500, showing an increasing trend in the intervention group and a decreasing trend in the control group. Participants who had more than 7500 steps per day at baseline may have had increased motivation levels when they first used the monitoring PHR app as they participated in the trial. Although we did not measure the levels of motivation, we assumed that the number of steps appeared to decline as the motivation levels decreased over time without additional rewards or interventions other than the text messages. Additionally, using the PHR app and monitoring the step counts at baseline may have had an impact on the results. Unblinded self-monitoring at baseline may result in high baseline step counts if participants gain insight into their step pattern, which may explain the gradual decrease in steps over time, as self-monitoring of behavior is an intervention in itself. Furthermore, if individuals were already walking more than 7500 steps per day, it may be challenging to increase their step count compared to those who walked less than 7500 steps per day. Regarding glycemic outcomes, in the intervention group, HbA1c at week 12 was significantly lower than that in the control group, and significant group differences were found in the changes in fasting blood glucose levels from baseline to week 12. There was a significant difference in the changes in body weight within the intervention group at weeks 12 and 24, with no significant difference between the groups. Physical activity tended to decrease at weeks 12 and 24 in the control group but increased slightly at week 12 and then decreased at week 24 in the intervention group. Individuals may encounter challenges in maintaining new behaviors before they become stable [21]. Altering and sustaining behavior is a complex process that involves both conscious and unconscious aspects [22]. Therefore, it is necessary to create multi-faceted interventions based on the personal aspect that can modify and maintain the healthy behaviors by providing necessary skills to do it themselves in future research.

According to the American Diabetes Association guidelines, patients with diabetes should perform at least 150 min of moderate-intensity aerobic exercise (walking) for 3–7 days a week without more than 2 days of rest per week [4]. A Previous RCT examined the effect of smartphone games on daily physical activity (steps/day) in inactive patients with type 2 diabetes, randomized into 18 patients in the intervention group and 17 patients in the control group [23]. The steps per day of intervention group was changed from 5,785 steps/day (pre-intervention) to 9,783 steps/day (post-intervention), the step per day of control group was changed from 5,612 steps/day to 6,552 steps/day, and the adjusted difference was significant (3,128 steps/day, 95% CI:2,313–3,943, *P* < 0.001). The study found that the intervention resulted in an increase in the number of steps taken by individuals with insufficient levels of physical activity, similar to the increase observed in patients who took less than 7500 steps per day in our study. A systematic review study found that smartphone app-based lifestyle modification interventions lowered HbA1c levels in patients with diabetes in several randomized controlled trials [10]. However, Thorsen IK and colleagues also investigated the effectiveness of including an app-based approach to increase moderate and vigorous physical activity compared with standard care among individuals with type 2 diabetes, but it did not increase physical activity as same as our study [11]. In this study, app-based programs were provided to both the intervention and control groups to control for the effectiveness of the app program itself, unlike previous studies that provided app-based programs only to the intervention group, and an encouraging message was sent only to the intervention group. Although we found that the overall number of steps decreased and the adjusted difference was not significant in the intervention and control groups, the number of steps per day of the intervention and control groups in participants with a baseline daily mean step of less than 7500 steps per day changed from 4,011 steps/day (pre-intervention) to 5,329 steps/day (post-intervention) and from 4,627 steps/day (pre-intervention) to 4,488 steps/day (post-intervention), respectively, with a significant group difference (*P* = 0.009). Unlike the previous studies, our study provided all participants with a smartphone PHR app and the number of daily steps at baseline was more than 7,000 steps, greater than those in the previous studies, which could weaken the effect of intervention in the total participants.

Given the glycemic outcomes, a meta-analysis of 47 RCTs that confirmed the blood glucose-lowering effect of exercise in type 2 diabetes patients found that structured exercise training, which lasted more than 150 min a week, significantly lowered HbA1c by 0.67% compared to the control group but reported that the only physical activity advice was not significant for changes in HbA1c [24]. In our study, the change in HbA1c between the two groups from baseline to 12 weeks was not significant; however, there was a significant mean difference in HbA1c levels at 12 weeks. Most of the studies included in the meta-analysis of physical activity advice alone had no limitation on HbA1c levels in inclusion criteria, with mean baseline HbA1c of 12 studies at 7.6%, while our study included patients with type 2 diabetes having less than HbA1c of 8.5%, and baseline HbA1c level of the current study was relatively low at 7.1 ± 0.4%, which could show weak intervention outcomes in glycemic control, as low baseline HbA1c level is associated with small magnitude of its change after medication treatment and intervention [25]. Based on these results, future studies should be conducted on patients with poor glycemic control, and more integrated and reward-enhancing interventions are needed, as shown to have a significant effect when dietary co-intervention is added to physical activity advice.

In this study, HbA1c and body weight decreased in both groups until 12 and 24 weeks, when the app was used in all participants. This indicates that the positive benefits of weight reduction and glycemic management can be observed with the use of the smartphone PHR app. Additionally, by receiving an encouraging text message on physical activity, the intervention group's improvements in HbA1c levels, fasting blood glucose levels, and body weight were identified. However, the degree of improvement achieved by app-based interventions may not be sufficient to yield great health benefits. Therefore, people with low level of physical activity or poor glycemic control may benefit from this app-based intervention and providing additional interventions may be help them improve. A meaningful finding of our study is that encouraging physical activity by sending text messages on steps per day is useful in patients with daily mean steps of less than 7500 steps per a day. Also encouraging strategy has a beneficial effect on glycemic control, especially in patients with baseline HbA1c ≥ 7.5%. Taken together, app-based interventions may be effective for patients with low levels of physical activity and uncontrolled glycemia.

This study has several strengths. This was a 12-week, randomized, controlled, and 12-week extension study with 200 participants, which had substantial data regarding the efficacy of smartphone PHR app-based interventions on physical activity and glycemic control in patients with type 2 diabetes. This study adopted a smartphone PHR app-based targeted encouraging text message for physical activity according to the mean number of steps per day in the previous week. In addition, since all participants received monitoring smartphone PHR apps and additional devices such as a blood glucometer, sphygmomanometer, and scale linked to the app, we were able to identify the interventional effect of encouraging text messages on physical activity and glycemic outcomes. A limitation of our study is that unblinded data were collected during the study period at baseline, week 12, and week 24. Second, as our study was conducted at a single center, the study population may not be representative of all Korean patients with type 2 diabetes. Also, our study population was restricted to patients with type 2 diabetes who had baseline less than HbA1c of 8.5% and BMI ≥ 23 kg/m^2^ and not use insulin, glucagon-like peptide 1 agonist. Thus, further studies are needed with more obese patients, those receiving injection treatment, and patients with poorly controlled type 2 diabetes. The subgroup analysis based on HbA1c levels of 7.5% and daily step counts of 7,500 was not pre-specified. Finally, while the mean difference of HbA1c at week 12 was statistically significant, it may not be clinically significant as the clinically meaningful reduction of HbA1c is considered to be 0.5%.

## Conclusions

App-based individualized motivational intervention for physical activity did not increase daily step count from baseline to week 12, and the changes in HbA1c levels from baseline to week 12 were comparable.

### Supplementary Information

**Supplementary Material 1.**

## Data Availability

Data supporting the conclusions of this study are included in the article and its additional files.

## Abbreviations

App: Application
BMI: Body mass index
CI: Confidence interval
CKD: Chronic kidney disease
DPP4: Dipeptidyl peptidase 4
eGFR: Estimated glomerular filtration rate
GLP-1: Glucagon like peptide-1
HbA1c: Glycated hemoglobin
HDL: High-density lipoprotein
IPAQ-SF: International Physical Activity Questionnaire-Short Form
IRB: Institutional Review Board
LDL: Low-density lipoprotein
METs: Metabolic equivalents of tasks
PHR: Personal health record
RCT: Randomized clinical trial
SD: Standard deviation
SGLT: Sodium/glucose cotransporter 2
SMC: Samsung Medical Center
T2D: Type 2 diabetes

## References

[1] Sun H, Saeedi P, Karuranga S, Pinkepank M, Ogurtsova K, Duncan BB, Stein C, Basit A, Chan JCN, Mbanya JC (2022). IDF Diabetes Atlas: Global, regional and country-level diabetes prevalence estimates for 2021 and projections for 2045. Diabetes Res Clin Pract.

[2] Jeon YK, Kim SS, Kim JH, Kim HJ, Kim HJ, Park JJ, Cho YS, Joung SH, Kim JR, Kim BH (2020). Combined aerobic and resistance exercise training reduces circulating Apolipoprotein j levels and improves insulin resistance in postmenopausal diabetic women. Diabetes Metab J.

[3] Lee YJ, Han KD, Kim JH (2022). Association among current smoking, alcohol consumption, regular exercise, and lower extremity amputation in patients with diabetic foot: nationwide population-based study. Endocrinol Metab (Seoul).

[4] Colberg SR, Sigal RJ, Yardley JE, Riddell MC, Dunstan DW, Dempsey PC, Horton ES, Castorino K, Tate DF (2016). Physical activity/exercise and diabetes: a position statement of the American Diabetes Association. Diabetes Care.

[5] Tudor-Locke C, Craig CL, Brown WJ, Clemes SA, De Cocker K, Giles-Corti B, Hatano Y, Inoue S, Matsudo SM, Mutrie N (2011). How many steps/day are enough? For adults. Int J Behav Nutr Phys Act.

[6] Boulé NG, Haddad E, Kenny GP, Wells GA, Sigal RJ (2001). Effects of exercise on glycemic control and body mass in type 2 diabetes mellitus: a meta-analysis of controlled clinical trials. JAMA.

[7] Schneider SH, Amorosa LF, Khachadurian AK, Ruderman NB (1984). Studies on the mechanism of improved glucose control during regular exercise in type 2 (non-insulin-dependent) diabetes. Diabetologia.

[8] Yamanouchi K, Shinozaki T, Chikada K, Nishikawa T, Ito K, Shimizu S, Ozawa N, Suzuki Y, Maeno H, Kato K (1995). Daily walking combined with diet therapy is a useful means for obese NIDDM patients not only to reduce body weight but also to improve insulin sensitivity. Diabetes Care.

[9] Gregg EW, Gerzoff RB, Caspersen CJ, Williamson DF, Narayan KM (2003). Relationship of walking to mortality among US adults with diabetes. Arch Intern Med.

[10] Lunde P, Nilsson BB, Bergland A, Kværner KJ, Bye A (2018). The effectiveness of smartphone apps for lifestyle improvement in noncommunicable diseases: systematic review and meta-analyses. J Med Internet Res.

[11] Thorsen IK, Yang Y, Valentiner LS, Glümer C, Karstoft K, Brønd JC, Nielsen RO, Brøns C, Christensen R, Nielsen JS (2022). The effects of a lifestyle intervention supported by the interwalk smartphone app on increasing physical activity among persons with type 2 diabetes: parallel-group Randomized Trial. JMIR Mhealth Uhealth.

[12] Kollins SH, DeLoss DJ, Cañadas E, Lutz J, Findling RL, Keefe RSE, Epstein JN, Cutler AJ, Faraone SV (2020). A novel digital intervention for actively reducing severity of paediatric ADHD (STARS-ADHD): a randomised controlled trial. Lancet Digit Health.

[13] Mitsui T, Shimaoka K, Tsuzuku S, Kajioka T, Sakakibara H (2008). Pedometer-determined physical activity and indicators of health in Japanese adults. J Physiol Anthropol.

[14] Inker LA, Eneanya ND, Coresh J, Tighiouart H, Wang D, Sang Y, Crews DC, Doria A, Estrella MM, Froissart M (2021). New Creatinine- and Cystatin C-based equations to estimate GFR without race. N Engl J Med.

[15] Craig CL, Marshall AL, Sjöström M, Bauman AE, Booth ML, Ainsworth BE, Pratt M, Ekelund U, Yngve A, Sallis JF, Oja P (2003). International physical activity questionnaire: 12-country reliability and validity. Med Sci Sports Exerc.

[16] Lee PH, Macfarlane DJ, Lam TH, Stewart SM (2011). Validity of the International Physical Activity Questionnaire Short Form (IPAQ-SF): a systematic review. Int J Behav Nutr Phys Act.

[17] Nishimoto D, Kodama S, Nishio I, Makizako H, Ku-Ohl Project T (2022). Association between the perception of behavior change and habitual exercise during COVID-19: a cross-sectional online survey in Japan. Int J Environ Res Public Health.

[18] Glynn LG, Hayes PS, Casey M, Glynn F, Alvarez-Iglesias A, Newell J, ÓLaighin G, Heaney D, O’Donnell M, Murphy AW (2014). Effectiveness of a smartphone application to promote physical activity in primary care: the SMART MOVE randomised controlled trial. Br J Gen Pract.

[19] Kirwan M, Duncan MJ, Vandelanotte C, Mummery WK (2012). Using smartphone technology to monitor physical activity in the 10,000 Steps program: a matched case–control trial. J Med Internet Res.

[20] Patel MS, Benjamin EJ, Volpp KG, Fox CS, Small DS, Massaro JM, Lee JJ, Hilbert V, Valentino M, Taylor DH (2017). Effect of a game-based intervention designed to enhance social incentives to increase physical activity among families: The BE FIT randomized clinical trial. JAMA Intern Med.

[21] Huttunen-Lenz M, Hansen S, Raben A, Westerterp-Plantenga M, Macdonald I, Stratton G, Swindell N, Martinez JA, Handjieva-Darlenska T, Poppitt SD (2022). Forming new health behavior habits during weight loss maintenance-The PREVIEW study. Health Psychol.

[22] Michaelsen MM, Esch T (2021). Motivation and reward mechanisms in health behavior change processes. Brain Res.

[23] Höchsmann C, Müller O, Ambühl M, Klenk C, Königstein K, Infanger D, Walz SP, Schmidt-Trucksäss A (2019). Novel smartphone game improves physical activity behavior in type 2 diabetes. Am J Prev Med.

[24] Umpierre D, Ribeiro PAB, Kramer CK, Leitão CB, Zucatti ATN, Azevedo MJ, Gross JL, Ribeiro JP, Schaan BD (2011). Physical activity advice only or structured exercise training and association with HbA1c levels in type 2 diabetes: a systematic review and meta-analysis. JAMA.

[25] DeFronzo RA, Stonehouse AH, Han J, Wintle ME (2010). Relationship of baseline HbA1c and efficacy of current glucose-lowering therapies: a meta-analysis of randomized clinical trials. Diabet Med.

