# Supplementary material for: A randomized controlled trial of an app-based intervention on physical activity and glycemic control in people with type 2 diabetes
Source: BMC Med. 2024 May 1;22:185. doi: 10.1186/s12916-024-03408-w (PMC11064293; doi:10.1186/s12916-024-03408-w)
Supplement: Supplementary file 1 — Supplementary Material 1. [file 12916_2024_3408_MOESM1_ESM.docx]

**A randomized controlled trial of an app-based intervention on physical activity and glycemic control in people with type 2 diabetes**

Gyuri Kim MD, PhD^1,^**^†^**, Seohyun Kim^2,^**^†^**, You-Bin Lee MD, PhD^1^, Sang-Man Jin MD, PhD^1^, Kyu Yeon Hur MD, PhD^1^, Jae Hyeon Kim MD, PhD^1,2,*^

^1^Division of Endocrinology and Metabolism, Department of Medicine, Samsung Medical Center, Sungkyunkwan University School of Medicine, Seoul 06351, Republic of Korea

^2^Department of Clinical Research Design and Evaluation, Samsung Advanced Institute for

Health Sciences and Technology, Sungkyunkwan University, Seoul 06355, Republic of Korea

**^†^**These two authors contributed equally to this work.

**Figure S1.** Flow chart

**Figure S2.** Changes in HbA1c at each follow up

**Figure S3.** Changes in HbA1c at each follow up (subjects with baseline HbA1c <7.5%, N=152)

**Figure S4.** Changes in HbA1c at each follow up (subjects with baseline HbA1c ≥7.5%, N=30)

**Table S1.** Results of linear mixed effect model

**Table S2.** Changes in fasting plasma glucose levels from baseline to week 12 and week 24

**Table S3.** Changes in body weight from baseline to week 12 and week 24

**Table S4.** Total MET-min/week at baseline, week 12, and week 24

**Table S5.** Changes in lipid levels from baseline to week 12 and week 24

**Figure S1.** Flow chart


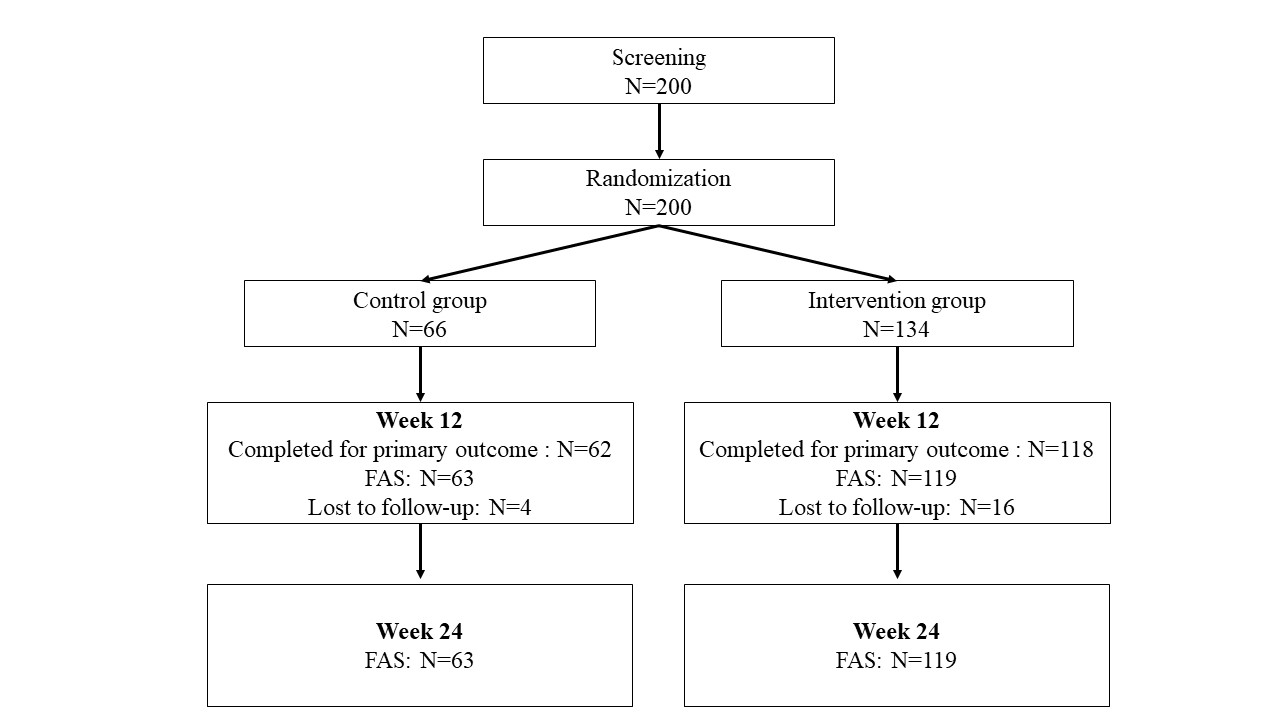


**Figure S2.** Changes in HbA1c at each follow up


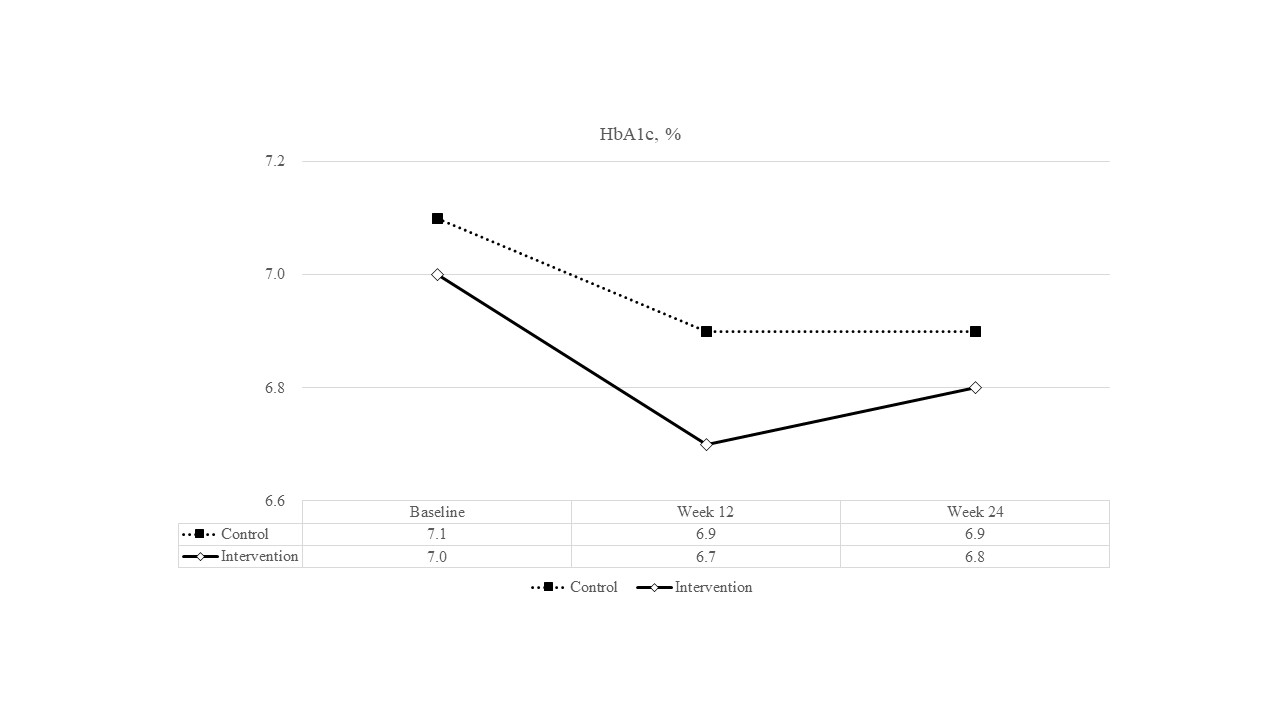


**Figure S3.** Changes in HbA1c at each follow up (subjects with baseline HbA1c <7.5%, N=152)


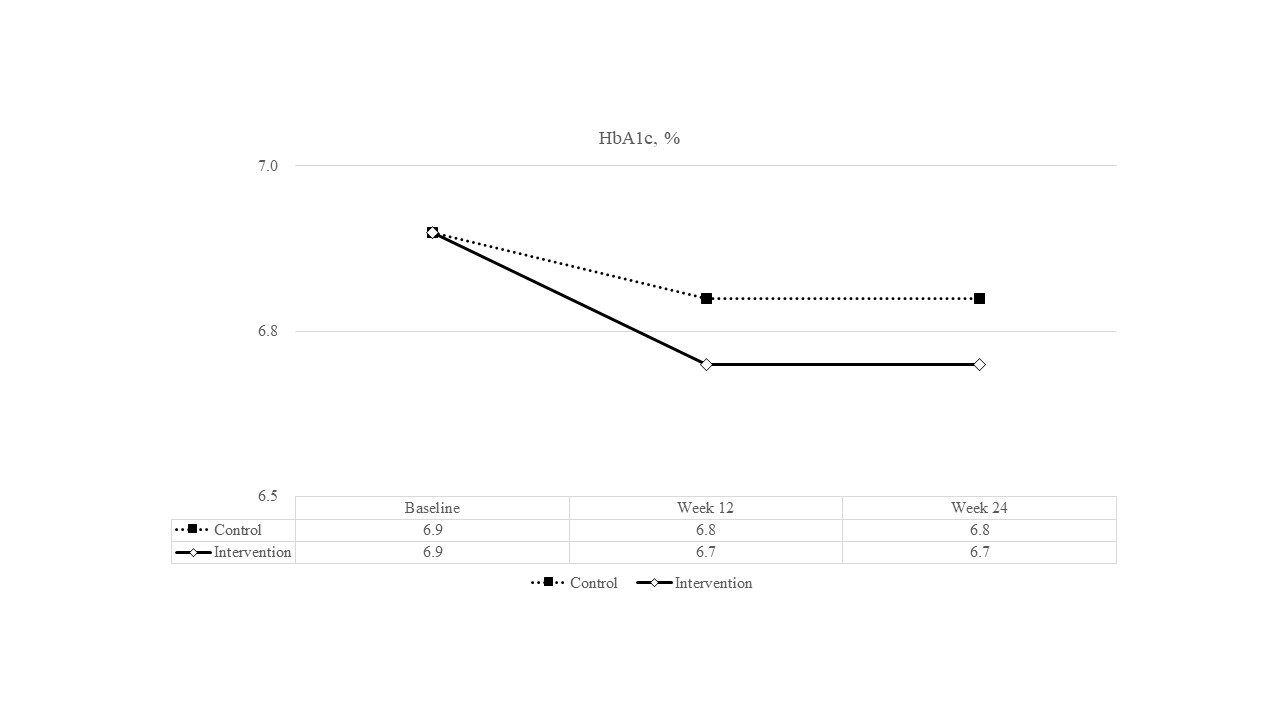


**Figure S4.** Changes in HbA1c at each follow up (subjects with baseline HbA1c ≥7.5%, N=30)


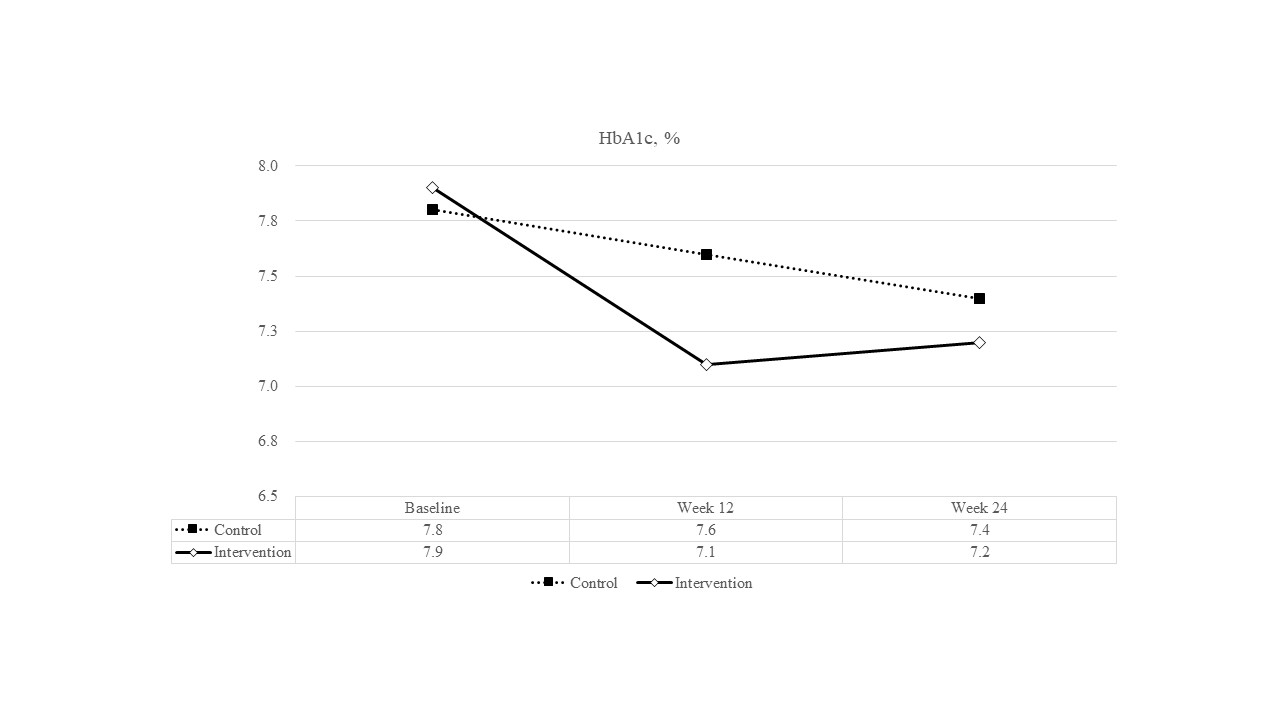


**Table S1. Results of linear mixed effect model**

|  | Control (β, 95% CI) | Intervention (β, 95% CI) | P value |
| --- | --- | --- | --- |
| No. of patients | 63 | 119 |  |
| Average difference of step counts in every 12 weeks | 0 (Reference) | 76 (642, -490) | 0.791 |
| Average difference of HbA1c (%) in every 12 weeks | 0 (Reference) | -0.05 (0.05, -0.14) | 0.329 |

* Abbreviations: CI, confidence interval

**Table S2. Changes in fasting plasma glucose levels from baseline to week 12 and week 24**

|  | **Control** | | **Intervention** | | **P-value** |
| --- | --- | --- | --- | --- | --- |
|  | N | FPG (mg/dL) | N | FPG (mg/dL) |  |
| Baseline (Mean ± SD) | 63 | 135.2 ± 21.1 | 119 | 138.2 ± 24.9 | ${0.421}^{b}$ |
| At week 12 (Mean ± SD) | 62 | 134.1 ± 22.7 | 117 | 128.3 ± 21.1 | ${0.090}^{b}$ |
| Changes from baseline to week 12 (Mean ± SD) | -0.84 ± 22.92 | | -9.22 ± 21.25 | | ${0.016}^{b}$ |
| P-value for mean difference from baseline to week 12 | ${0.774}^{a}$ | | <${0.001}^{a}$ | |  |
| At week 24 (Mean ± SD) | 62 | 136.4 ± 22.5 | 117 | 133.6 ± 25.7 | ${0.475}^{b}$ |
| Changes from baseline to week 24 (Mean ± SD) | 1.44 ± 19.29 | | -3.91 ± 25.41 | | ${0.148}^{b}$ |
| P-value for mean difference from baseline to week 24 | ${0.560}^{a}$ | | ${0.147}^{a}$ | |  |

Abbreviations: FPG, fasting plasma glucose; SD, standard deviation

${}^{a}$P-values were derived using paired t-tests.

${}^{b}$P-values were derived from a two-sample t-test.

**Table S3. Changes in body weight from baseline to week 12 and week 24**

|  | **Control** | | **Intervention** | | **P-value** |
| --- | --- | --- | --- | --- | --- |
|  | N | Weight (kg) | N | Weight (kg) |  |
| Baseline (Mean ± SD) | 63 | 73.5 ± 8.6 | 119 | 73.7 ± 9.8 | ${0.898}^{b}$ |
| At week 12 (Mean ± SD) | 62 | 72.4 ± 8.7 | 118 | 72.8 ± 9.5 | ${0.821}^{b}$ |
| Changes from baseline to week 12 (Mean ± SD) | -0.65 ± 3.62 | | -1.07 ± 2.06 | | ${0.846}^{b}$ |
| P-value for mean difference from baseline to week 12 | ${0.161}^{a}$ | | <${0.001}^{a}$ | |  |
| At week 24 (Mean ± SD) | 58 | 71.6 ± 8.9 | 109 | 72.4 ± 9.8 | ${0.324}^{b}$ |
| Changes from baseline to week 24 (Mean ± SD) | -1.05 ± 3.62 | | -1.46 ± 2.22 | | ${0.370}^{b}$ |
| P-value for mean difference from baseline to week 24 | ${0.030}^{a}$ | | <${0.001}^{a}$ | |  |

Abbreviations: SD, standard deviation

${}^{a}$P-values were derived using paired t-tests.

${}^{b}$P-values were derived from a two-sample t-test.

**Table S4. Total MET-min/week at baseline, week 12, and week 24**

|  | **Control** | | **Intervention** | | **P-value** |
| --- | --- | --- | --- | --- | --- |
|  | N | Weight (kg) | N | Weight (kg) |  |
| Total MET-min/week at baseline | 61 | 2123.4 ± 2539.6 | 117 | 1792.1 ± 2648.3 | ${0.423}^{a}$ |
| Total MET-min/week at week 12 | 61 | 1824.6 ± 1867.9 | 115 | 1906.9 ± 2273.8 | ${0.809}^{a}$ |
| Total MET-min/week at week 24 | 39 | 1874.6 ± 2157.4 | 76 | 1578.5 ± 2775.2 | ${0.562}^{a}$ |

* Abbreviations: MET, metabolic equivalent of task; SD, standard deviation

${}^{a}$P-values were derived using paired t-tests.

**Table S5. Changes in lipid levels from baseline to week 12 and week 24**

|  | **Control** | | **Intervention** | | | **P-value** | |  |
| --- | --- | --- | --- | --- | --- | --- | --- | --- |
|  | N | Mean ± SD (mg/dL) | | N | Mean ± SD (mg/dL) | |  | |
| **Total cholesterol** |  |  | |  |  | |  | |
| Baseline | 63 | 142.8 ± 27.2 | | 116 | 147.6 ± 29.4 | | ${0.281}^{a}$ | |
| At week 12 | 57 | 142.1 ± 29.2 | | 105 | 144.8 ± 25.4 | | ${0.542}^{a}$ | |
| At week 24 | 28 | 144.8 ± 28.3 | | 59 | 143.2 ± 27.0 | | ${0.796}^{a}$ | |
| **LDL cholesterol** |  |  | |  |  | |  | |
| Baseline | 63 | 81.8 ± 25.6 | | 117 | 86.7 ± 28.7 | | ${0.256}^{a}$ | |
| At week 12 | 57 | 81.3 ± 26.0 | | 105 | 85.5 ± 26.2 | | ${0.329}^{a}$ | |
| At week 24 | 27 | 84.1 ± 24.3 | | 55 | 85.0 ± 26.0 | | ${0.878}^{a}$ | |
| **HDL cholesterol** |  |  | |  |  | |  | |
| Baseline | 63 | 55.1 ± 14.1 | | 117 | 50.6 ± 13.7 | | ${0.036}^{a}$ | |
| At week 12 | 57 | 55.2 ± 13.0 | | 105 | 53.3 ± 12.8 | | ${0.355}^{a}$ | |
| At week 24 | 27 | 55.6 ± 13.4 | | 55 | 52.5 ± 13.3 | | ${0.334}^{a}$ | |
| **Triglycerides** |  |  | |  |  | |  | |
| Baseline | 63 | 131.1 ± 63.7 | | 117 | 154.8 ± 140.7 | | ${0.123}^{a}$ | |
| At week 12 | 57 | 121.8 ± 57.8 | | 105 | 130.7 ± 84.1 | | ${0.425}^{a}$ | |
| At week 24 | 28 | 122.0 ± 46.0 | | 55 | 127.3 ± 71.0 | | ${0.682}^{a}$ | |

Abbreviations HDL, high-density lipoprotein; LDL, low-density lipoprotein; SD, standard deviation

${}^{a}$P-values were derived from a two-sample t-test.
